# Laminin-rich blood vessels display activated growth factor signaling and act as the proliferation centers in Dupuytren’s contracture

**DOI:** 10.1186/s13075-015-0661-y

**Published:** 2015-05-28

**Authors:** Janeli Viil, Katre Maasalu, Kristina Mäemets-Allas, Liis Tamming, Kadi Lõhmussaar, Mikk Tooming, Sulev Ingerpuu, Aare Märtson, Viljar Jaks

**Affiliations:** Institute of Molecular and Cell Biology, University of Tartu, Riia 23, 51010 Tartu, Estonia; Department of Traumatology and Orthopedics, Tartu University Hospital, Puusepa 8, 51014 Tartu, Estonia; Clinic of Traumatology and Orthopedics, Tartu University Hospital, Puusepa 8, 51014 Tartu, Estonia; Department of Biosciences and Nutrition, Karolinska Institutet, Hälsovägen 7, 141 83 Huddinge, Sweden

## Abstract

**Introduction:**

Dupuytren’s contracture (DC) is a chronic fibroproliferative disease of the hand, which is characterized by uncontrolled proliferation of atypical myofibroblasts at the cellular level. We hypothesized that specific areas of the DC tissue are sustaining the cell proliferation and studied the potential molecular determinants that might contribute to the formation of such niches.

**Methods:**

We studied the expression pattern of cell proliferation marker Ki67, phosphorylated AKT (Ak mouse strain thymoma) kinase, DC-associated growth factors (connective tissue growth factor (CTGF), basic fibroblast growth factor (bFGF), insulin-like growth factor 2 (IGF-2)) and extracellular matrix components (laminins, fibronectin, collagen IV) in DC tissue and normal palmar fascia using immunofluorescence microscopy and quantitative real-time polymerase chain reaction (qPCR).

**Results:**

We found that proliferative cells in the DC nodules were concentrated in the immediate vicinity of small blood vessels and localized predominantly in the myofibroblast layer. Correspondingly, the DC-associated blood vessels contained increased levels of phosphorylated AKT, a hallmark of activated growth factor signaling. When studying the expression of potential activators of AKT signaling we found that the expression of bFGF was confined to the endothelium of the small blood vessels, IGF-2 was present uniformly in the DC tissue and CTGF was expressed in the DC-associated sweat gland acini. In addition, the blood vessels in DC nodules contained increased amounts of laminins 511 and 521, which have been previously shown to promote the proliferation and stem cell properties of different cell types.

**Conclusions:**

Based on our findings, we propose that in the DC-associated small blood vessels the presence of growth factors in combination with favorable extracellular matrix composition provide a supportive environment for sustained proliferation of myofibroblasts and thus the blood vessels play an important role in DC pathogenesis.

**Electronic supplementary material:**

The online version of this article (doi:10.1186/s13075-015-0661-y) contains supplementary material, which is available to authorized users.

## Introduction

Dupuytren’s contracture (DC) is a chronic, progressive fibroproliferative disease of the hand, which affects 4 to 11 % of general population [1, 2]. It is widely accepted that uncontrolled proliferation of atypical fibroblasts forms the cellular basis of DC. Nodules, which are formed by proliferative myofibroblasts and contain an increased number of small blood vessels, appear in the early stages of the disease [3]. As DC progresses differentiation of fibroblasts leads to formation of thickened collagen-rich cords, which cause flexion deformity of the affected fingers and a severe reduction in hand function [1, 4]. Clinically, DC is reminiscent of a benign dysplastic disorder. It does not disseminate to other tissues but can invade locally and has a progressive and irreversible course [5].

Despite the extensive research addressing this disease, the exact etiology and pathogenetic mechanisms of DC remain largely unknown. A complex network of events, including deregulation of cytokine and growth factor signaling along with changes in the extracellular matrix (ECM) components (e.g., collagens and laminins), is implicated in the progression of the DC [6, 7]. Systemic analysis of the proteome and transcriptome of the DC cells have shown that aberrant activation of Ak mouse strain thymoma-associated (AKT) kinase, mitogen-activated protein kinase (MAPK) and transforming growth factor β (TGF-β) pathways play a prominent role in DC pathogenesis by inducing cell proliferation and fibrosis correspondingly [3, 8, 9]. A few growth factors, which are thought to activate the AKT and MAPK pathways in DC, have been identified. It has been shown that DC tissue contains increased amounts of basic fibroblast growth factor (bFGF) [10]. Furthermore, cultured DC fibroblasts have shown to synthetize connective tissue growth factor (CTGF) [9, 11] and insulin-like growth factor 2 (IGF-2) [12], which sustained their proliferation *in vitro*.

Laminins (LM), the main components of basal membrane (BM) along with collagen IV (CoIV), belong to a well-characterized family of trimeric glycoproteins and consist of one α, one β and one γ subunit [13]. Laminins interact with adhesion molecules on the cell surface and modulate intracellular signaling pathways including those related to multipotency and stemness [14–16]. The laminin isoform composition in the DC has been addressed before [7, 17]; however, these studies were limited by the availability of antibodies specific to certain laminin subunits.

In this study, we hypothesized that specific areas of the DC tissue are sustaining the cell proliferation and studied the potential molecular determinants that might contribute to the formation of such niches.

We found that the proliferating cells were concentrated in the immediate vicinity of the DC-associated blood vessels, which contained increased amount of bFGF, phosphorylated AKT (pAKT) protein and laminins 511 and 521. Our findings suggest the presence of a supportive microenvironment in the immediate vicinity of small blood vessels in the DC tissue, which sustains the persistent myofibroblast proliferation and promotes the progression of this disease.

## Methods

### Ethics statement

The procedures for sample collection and processing were approved by the Research Ethics Committee of the University of Tartu (permit no 221/M-34). Written informed consent was obtained from the research subjects.

### DC samples

DC samples were obtained from biopsies taken from eight DC patients with extensive, stage 2 or 4 fibrosis of the palmar fascia during open radical palmar fasciectomy. The nodular tissue was separated from the DC chords and embedded in Tissue-Tek O.C.T. Compound (Sakura Finetek, Torrance, CA, USA). The embedded samples were subsequently shifted to −80 °C until further use. Samples of normal palmar fascia were obtained from four patients during the open carpal tunnel release operations and were used as controls.

### Immunostaining and imaging

Frozen tissue sections (10–14 μm thick) were fixed with 4 % formaldehyde for 10 minutes, permeabilized with 0.1% Triton X for 10 minutes and blocked with 4 % normal goat or donkey serum in phosphate-buffered saline (PBS) for 1 hour. Next, the tissue sections were incubated with primary antibodies for 1 hour, followed by the incubation of fluorochrome-labeled secondary antibodies for 45 minutes (1:500, Life Technologies, Carlsbad, CA, USA).

For staining with two mouse monoclonal antibodies the staining with the first antibody was performed as described above. Next, the sections were blocked with M.O.M. (Mouse-On-Mouse) Mouse Ig Blocking Reagent (Vector Laboratories, Burlingame, CA, USA) for 1 hour, followed by the incubation with M.O.M. Diluent (Vector Laboratories) for 5 minutes. Sections were then incubated with the second set of primary and secondary antibodies diluted in M.O.M. Diluent for 1 hour.

When co-staining with anti-CD105-fluorescein isothiocyanate (FITC) conjugate was performed, the sections were initially stained with the first set of primary and secondary antibodies as described, then the sections were blocked with 4 % rabbit serum in PBS containing nonspecific mouse immunoglobulins (1/100) for 1 hour. Next, the sections were incubated with CD105-FITC conjugate for 1 hour and subsequently with rabbit anti-FITC Alexa Fluor 488-conjugated secondary antibody for 45 minutes (1:500, Life Technologies) to enhance the CD105 signal.

The primary antibodies used in this study are listed in Table S1 in Additional file 1. Cell nuclei were visualized with DAPI (1 μg/ml, Sigma-Aldrich, St Louis, MO, USA) and sections were mounted with Fluorescent Mounting Medium (Dako, Glostrup, Denmark). All staining procedures were performed at room temperature.

The images were captured with Olympus BX61 and IX81 fluorescence microscopes or Olympus confocal microscope FV1000 (Olympus, Tokyo, Japan) and processed using Hokawo v2.1 (Hamamatsu Photonics, Hamamatsu, Japan) or Imaris (Bitplane AG, Zurich, Switzerland) software.

### Quantitative real-time PCR (qPCR)

Tissue samples were immersed in RNAlater (Thermo Fisher Scientific, Waltham, MA, USA) and stored at 4 °C for later analysis. The tissue was homogenized in 1 ml of TRIzol (Gibco, Carlsbad, CA, USA) by using Precellys®24 high-throughput tissue homogenizer (Precellys, Montigny-le-Bretonneux, France). The homogenate was then mixed with 0.2 ml of chloroform and centrifuged for 15 minutes at 12,000 × g (4 °C). The aqueous phase was transferred to a fresh tube, and the total RNA was extracted. One microgram of total RNA was reverse transcribed with RevertAid First Strand cDNA Synthesis Kit (Thermo Fisher Scientific) according to manufacturer’s instructions. The complementary DNA (cDNA) from each sample was loaded in triplicate onto a plate with primers (10 nM) and Maxima SYBR Green/ROX qPCR Master Mix reagents (Thermo Fisher Scientific). All qPCR reactions were carried out with an ABI Prism 7900 HT Sequence Detection System (Applied Biosystems Carlsbad, CA, USA), and the data acquired were analyzed with ABI SDS software (version 2.1 from Applied Biosystems). Housekeeping genes HPRT1 and RPLP0 were used as references. The following primers were used for the qPCR analysis: FGF2 5′-CTGGCTATGAAGGAAGATGGA-3′, 5′-TGCCCAGTTCGTTTCAGTG-3′, CTGF 5′-TGCGAGGAGTGGGTGTGTGA-3′, 5′-ACAGGTCTTGGAACAGGC-3′, IGF 5′-CCGTGCTTCCGGACAACTT-3′, 5′-TGGACTGCTTCCAGGTGTCA-3′, HPRT1 5′-GCCCTGGCGTCGTGATTAG-3′, 5′-ATAGCCCCCCTTGAGCACACA-3′, RPLP0 5′-TGCAGATTGGCTACCCAA-3′, 5′-TTCAGCAAGTGGGAAGGT-3′. The PCR reactions were performed at least three times for each sample and the average value of the reads was calculated. The box plots were generated using a free online tool [18].

## Results

### Blood vessels are proliferation epicenters in the DC

**Fig. 1:**
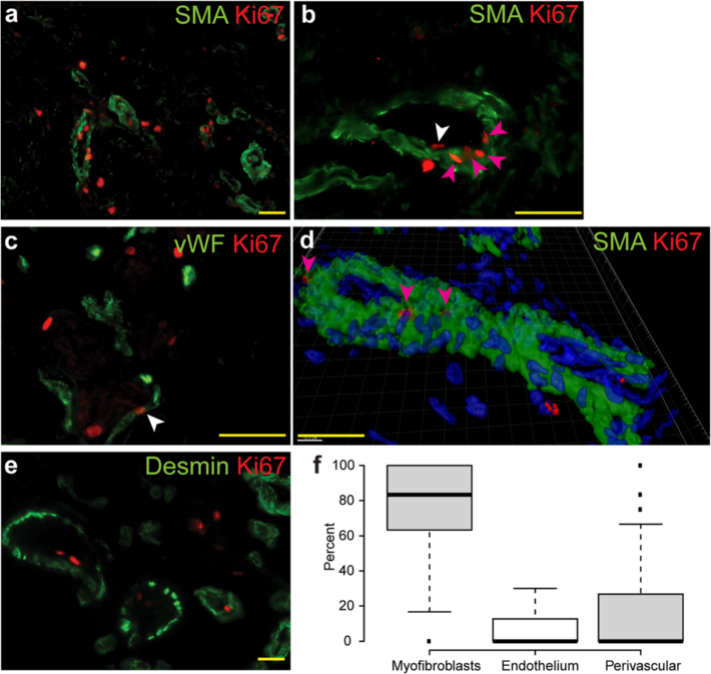
Proliferative cells locate prevalently in the myofibroblast compartment of small blood vessels in DC. Sections were stained with antibodies recognizing SMA, vWF, desmin and Ki67 as indicated on the panels. Proliferative Ki67-positive cells concentrate around and inside the small blood vessels in DC (**a**) and are located prevalently in the SMA-positive myofibroblast compartment of the blood vessels (**b, d**, *violet arrows*). (**d**) represents a surface-rendering model of an image stack obtained with a confocal microscope. Proliferative nuclei (*violet arrows*) are embedded in the SMA-expressing myofibroblast layer (*green*). Rare Ki67-positive cells were found in the vWF-positive endothelial compartment (**c**, *white arrows*), which is located inside the SMA-stained myofibroblast layer (**b**, *white arrow*). No proliferative cells could be detected among the desmin-labeled pericytes (**e**). Quantitation of the relative number of proliferative cells inside and in the immediate vicinity of the blood vessel wall (**f**, 6 patients, 45 sections) reveals that the majority of the proliferative cells are found in the myofibroblast layer. Scale bars: 20 μm. *DC* Dupuytren’s contracture, *SMA* smooth muscle actin, *vWF* von Willebrand’s factor

To investigate in detail the distribution of proliferative cells in the DC tissue we stained the DC and normal palmar fascia (NPF) samples with antibodies recognizing the proliferation marker Ki67 and the myofibroblast marker smooth muscle actin (SMA). Numerous Ki67-positive proliferating cells were identified in the DC tissue (Fig. 1a) whereas we could not detect any Ki-67-labeled cells in the NPF samples (Figure S1a in Additional file 2). Notably, the Ki67-positive proliferating cells were concentrated in the vicinity and inside of the SMA-expressing blood vessels (Fig. 1a). The identity of the SMA-positive structures as blood vessels was verified by co-staining the DC samples with SMA and endothelial marker von Willebrand’s factor (vWF) (Figure S1b in Additional file 2). As expected, we found that the DC nodules exhibited an increased overall SMA content when compared to NPF samples (Figure S1c, d in Additional file 2). The reason for this was an increased blood vessel number and the prevalence of SMA-positive myofibroblasts in DC tissue.

To study the proliferative activity of blood vessel wall components, we additionally co-stained the DC samples with antibodies recognizing vWF, pericyte marker desmin and Ki67. Interestingly, a vast majority of Ki-67-positive nuclei could be found in the strongly SMA-positive muscular layer of the blood vessels (Fig. 1b, d). However, only a minor part of the proliferative cells were mapped to the vWF-expressing endothelial compartment (Fig. 1c) and no proliferative desmin-positive pericytes could be identified (Fig. 1e). This was confirmed by quantitation of the relative number of proliferative cells in the endothelial, muscular and perivascular areas (Fig. 1f) by counting the proliferative nuclei in three different blood vessel compartments: in the SMA-expressing myofibroblast layer, the endothelial compartment, which is surrounded by the SMA-positive layer and the perivascular layer located outside of the SMA-positive layer. The obtained data confirmed our findings that the majority of the proliferating cells are found in the myofibroblast layer of the blood vessel wall.

### DC-associated blood vessels display an activated phenotype and contain CD105/CD90 double-positive endothelial cells

Since we saw an increase in the numbers of proliferating cells near the blood vessels in the DC tissue, we asked whether activated growth factor signaling might be present in the DC-associated blood vessels.

**Fig. 2:**
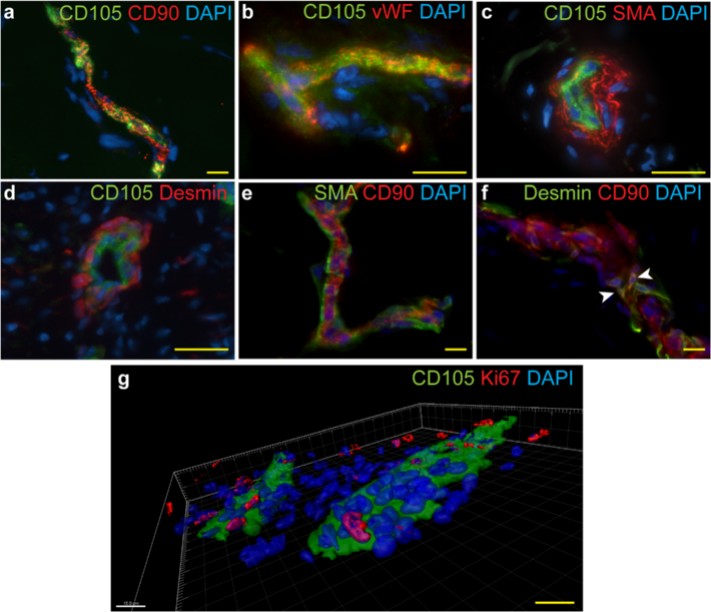
CD105 and CD90 label endothelial cells in the DC tissue. DC samples were stained with antibodies recognizing CD90, CD105, vWF, SMA and desmin as indicated on the panels. CD105/CD90 double-positive cells localized in blood vessels in the DC tissue (**a**). CD105 is localized in vWF-expressing endothelial cells (**b**). No CD105-expressing cells can be identified in the SMA-positive myofibroblast layer encompassing CD105-positive endothelial cells (**c**). Desmin-positive pericytes surround vWF-expressing endothelial cells and no CD105/desmin double-positive cells can be identified (**d**). No CD90/SMA double-positive myofibroblasts could be identified (**e**). Desmin-positive pericytes surround CD90-expressing cells with a few desmin/CD90 double-positive cells (**f**, *arrows*). Ki67-expressing proliferative cells are concentrated in the vicinity of CD105-expressing activated endothelium (**g**). Scale bars: 20 μm. *DC* Dupuytren’s contracture, *SMA* smooth muscle actin, *vWF* von Willebrand’s factor

To test our hypothesis we stained the tissue samples with antibodies recognizing CD90 (Thy-1) and CD105 (endoglin), as the expression of these molecules has been associated with the activated state of the endothelial cells in response to growth factor signaling [19, 20]. We detected a number of CD105/CD90 double-positive cell clusters located in the DC tissue (Fig. 2a, Figure S1e in Additional file 2). To verify the endothelial localization of CD105 we co-stained the DC samples with antibodies recognizing CD105 and vWF. As expected, CD105 was located in the vWF-positive endothelial cells (Fig. 2b, Figure S1f in Additional file 2). Double-staining of the DC samples with CD105 and SMA or desmin did not reveal any CD105/SMA or CD105/desmin double-positive cells and confirmed the endothelial localization of the CD105 expression (Fig. 2c, d). Notably, the rare small blood vessels found in NPF did not contain CD105-expressing cells (Figure S1g in Additional file 2). As expected, SMA-expressing myofibroblasts surrounded the CD90-expressing endothelial compartment and did not show any CD90 positivity (Fig. 2e, Figure S1h in Additional file 2). Similarly, the desmin-expressing pericyte compartment, which enclosed the CD90-expressing endothelium, was generally devoid of CD90-expressing cells and only very rare desmin/CD90 double-positive pericytes could be identified in the blood vessel wall (Fig. 2f). The endothelium of the vessels found in NPF samples, however, only very rarely contained CD90-expressing cells (Figure S1i in Additional file 2). As expected, the proliferating cells were located in the close proximity of the CD105-expressing activated endothelium in the DC samples, which is in good correlation with our previous results (Fig. 2g, see also Fig. 1a, b, d).

### The blood vessels in the DC tissue contain cells with activated AKT signaling

**Fig. 3:**
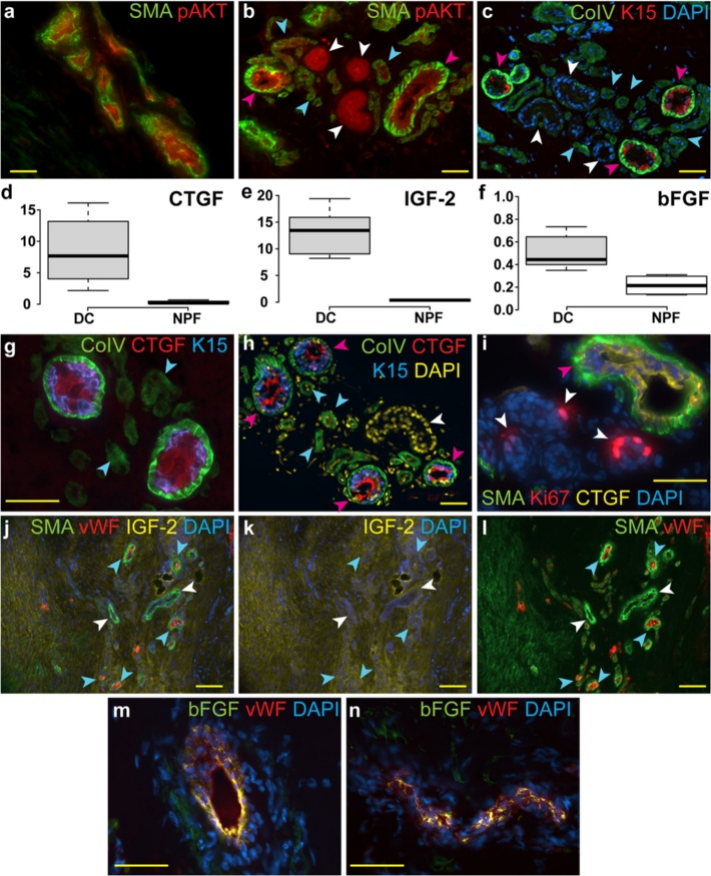
AKT pathway activation and the expression of CTGF, IGF-2 and bFGF in the DC tissue. DC samples were stained with antibodies recognizing SMA, phosphorylated AKT at T308 (pAKT), collagen IV (CoIV), keratin15 (K15), CTGF, IGF-2, bFGF and Ki67 as indicated on the panels. Nuclei were counterstained with DAPI. pAKT was detected in blood vessels (**a,b**; *blue arrows*), sweat gland acini (**b**; *violet arrows*) and sweat gland ducts (**b**; *white arrows*). Sweat gland acini were identified as containing characteristic K15-positive basal epithelium, which is encompassed by CoIV-positive basement membrane (BM; **c**; *violet arrows*). Sweat gland ducts (**c**; *white arrows*) and blood vessels (**c**; *blue arrows*) are negative for K15 and contain weakly CoIV-positive BM. The expression of CTGF (**d**), IGF-2 (**e**) and bFGF (**f**) was detected using qPCR in DC (n = 8) and NPF (n = 4) samples. Center lines show the medians; box limits indicate the 25th and 75th percentiles; whiskers extend 1.5 times the interquartile range from the 25th and 75th percentiles. The expression of all three growth factors was increased in DC samples when compared to NPF. K15-positive sweat gland acini synthetize CTGF (**g, h**; *violet arrows*) whereas ducts (**h**; *white arrows*) and blood vessels (**g, h**; *blue arrows*) are negative for CTGF. Sweat gland ducts contain dividing Ki67-positive cells (**i**; *white arrows*) while CTGF-expressing SMA-positive acini are not proliferative (**i**; *violet arrow*). IGF-2 is expressed throughout the DC tissue (**j-l**). The color-split images of (**j**) for IGF-2/DAPI (**k**) and SMA/vWF (**l**) are presented to underline the uniform distribution of IGF-2 expression in the DC tissue. bFGF co-localizes with vWF and thus is expressed in the endothelium of the DC-associated blood vessels as presented by cross (**m**) and longitudinal sections (**n**) of blood vessels. Scale bars: 50 μm. *AKT* Ak mouse strain thymoma-associated, *bFGF* basic fibroblast growth factor, *CTGF* connective tissue growth factor, *DC* Dupuytren’s contracture, *IGF-2* insulin-like growth factor 2, *NPF* normal palmar fascia, *qPCR* quantitative polymerase chain reaction, *SMA* smooth muscle actin

Since AKT pathway has been implicated in the pathogenesis of the DC [8], we hypothesized that activated AKT signaling might be involved in driving the enhanced proliferation in the vascular/perivascular compartment. To study the activation status of the AKT pathway we co-stained the DC samples with antibodies recognizing an activated and phosphorylated AKT (pAKT) isoform. We could readily detect the presence of pAKT in all the layers of SMA-positive blood vessels and their immediate vicinity (Fig. 3a). Interestingly, we did detect a high level of pAKT also in the acini and ducts of sweat glands (Fig. 3b), which were surrounded by small blood vessels. The identity of the sweat glands was verified by the keratin 15 (K15) and basement membrane marker CoIV staining (Fig. 3c). The K15-positive acinar basal epithelium of sweat glands is surrounded by CoIV-rich basement membrane, in contrast the ducts are negative for K15 and show less intensive CoIV staining similar to the surrounding blood vessels (Fig. 3c).

Since CTGF, bFGF and IGF-2 activate AKT kinase and have been implicated in the pathogenesis of DC [9, 10, 12, 21] we set out to investigate their expression in the NPF and DC samples. We found that the mRNA expression of all the three analyzed growth factors was increased in the DC samples when compared to NPF (Fig. 3d–f).

When probing the DC tissue sections with anti-CTGF antibody a strong signal was detected in the K15-positive acini of the sweat glands (Fig. 3g). CTGF expression was absent in other parts of the DC tissue including K15-negative sweat gland ducts and blood vessels (Fig. 3h). This suggests a hitherto unknown role for sweat glands in the pathogenesis of DC. Despite the activated pAKT signaling present both in the acini and ducts, the proliferative cells were located mainly in the sweat gland ducts while the CTGF-secreting cells in the acini did not show Ki67 positivity (Fig. 3i). The expression of IGF-2 was uniformly distributed over the DC tissue without any preference in respect of small blood vessels (Fig. 3j–l). Interestingly, the expression of bFGF was confined to the endothelium of the small blood vessels (Fig. 3m, n) correlating well with the increased number of proliferative cells in and near the blood vessels.

### Laminins 411/421, 511/521 and fibronectin are associated with the enhanced proliferation in the DC-associated blood vessels

Next, we asked whether specific components of the ECM might be present in the areas of DC tissue with increased proliferation. To describe in detail the constitution of the ECM in DC tissue, we stained tissue samples with antibodies recognizing human laminin subunits α2-α5, β1-β3, γ1-γ3, CoIV and fibronectin (FN).

**Fig. 4:**
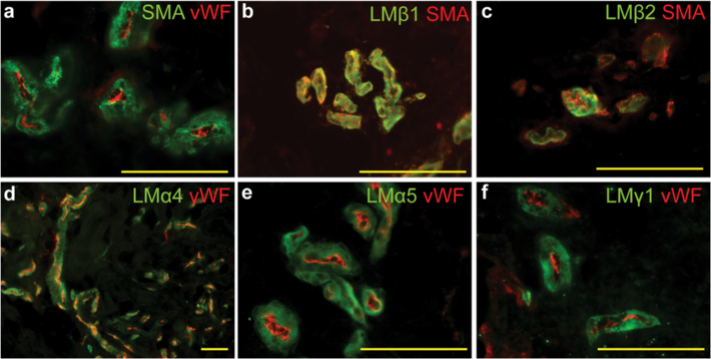
Laminins 411/421 and 511/521 localize in the small blood vessels in DC. The DC samples were stained with antibodies recognizing laminin subunits α4, α5, β1, β2 and γ1; vWF and SMA as indicated on the panels. SMA-labeled myofibroblast layer surrounds the vWF-positive endothelium in the blood vessels found in the DC tissue (**a**). Laminin subunits β1, β2, α4, α5 and γ1 (**b, c, d, e** and **f**) localize in the SMA- (**b, c**) or vWF-labeled (**d, e, f**) small blood vessels. Scale bars: 100 μm. *DC* Dupuytren’s contracture, *SMA* smooth muscle actin, *vWF* von Willebrand’s factor

The expression of laminin subunits α4, α5, β1, β2, and γ1 was detected in the DC tissue (Figure S2a-e in Additional file 3) but not in the NPF samples (Figure S2f-j in Additional file 3). However, we were not able to detect the expression of laminin subunits α2, α3, β2, β3, γ2 and γ3 neither in DC tissue (Figure S3a-d in Additional file 4) nor in NPF samples (Figure S3e-h in Additional file 4). The obtained data suggest that laminins 411, 421, 511 and 521 are contained in DC nodules. Since these laminin isoforms are expressed in vascular basement membranes [22] we co-stained the samples with antibodies specific for vWF or SMA and laminin subunits α4, α5, β1, β2, and γ1 (Fig. 4). The studied laminin subunits co-localized with the SMA-expressing myoepithelial cells (β1, β2: Fig. 4b, c) and surrounded the vWF positive endothelium (α4, α5, γ1: Fig. 4d, e, f) showing their localization in the myoepithelial compartment of the blood vessel wall. The laminin subunits α4, α5, β1, β2 and γ1 co-localized also with CoIV (Figure S3i-m in Additional file 4), which suggests that assembled basement membrane is present in the myoepithelial compartment of the blood vessel wall. Thus, the lack of laminins in NPF samples could be explained with the scarcity of blood vessels in NPF.

Fibronectin (FN) was widely expressed in both DC and NPF samples. In agreement with previous reports [23], the FN amount in DC was increased when compared to NPF samples (Figure S3n, o in Additional file 4) and the signal was more intensive in the laminin-positive blood vessels (compare Figure S3o, p in Additional file 4).

## Discussion

Despite the widespread nature of Dupuytren’s contracture, knowledge regarding the cellular and molecular mechanisms underlying the disease is still fragmentary. It is a widely accepted view that uncontrolled proliferation and accumulation of contractile myofibroblasts underlies the pathogenesis of DC [3]. In this work we sought to shed light on the molecular and structural determinants that sustain this process.

When studying the distribution of the proliferative cells in the DC tissue we found that these were concentrated in the small blood vessels or in their immediate vicinity. It has been shown previously that small blood vessels can act as stem cell niches in normal and malignant brain tissue [24, 25]. Our results suggest a comparable role for small blood vessels in DC, which might provide a favorable microenvironment for sustaining myofibroblast proliferation.

When looking for the cells positive for mesenchymal stem cell markers we found that endothelial cells in the blood vessels of DC nodules were double-positive for CD90 and CD105. CD90 is expressed on the activated endothelial cells in an inflammatory tissue and is necessary for the attachment of leukocytes to the endothelium ([26] and references therein). CD105 is found in the endothelium of immature blood vessels of developing tissues and in blood vessels associated with malignant tumors [27–29]. Thus, the expression of CD90 and CD105 in the blood vessels of the DC tissue correlates well with the presence of an inflammatory process associated with enhanced cell proliferation. Indeed, proteomic studies have put forward the proproliferative AKT signaling pathway, which is activated in response to growth factor stimulation, as a prominent player in DC pathogenesis [8]. In agreement with this notion, we detected elevated levels of phosphorylated AKT, a hallmark of activated AKT signaling, in the blood vessels and sweat glands within the DC nodule. Interestingly, the endothelial cells and the desmin-positive pericytes showed little response to the presence of proliferative signals. In contrast the SMA-expressing myofibroblasts present in the perivascular compartment and in the blood vessel wall contained a number of proliferative cells positive for the proliferation marker Ki67, showing that myofibroblasts are the most responsive cells to the proproliferative signaling present in the DC tissue. However, whether the proliferative myofibroblasts present in the blood vessel wall contribute substantially to the bulk of the DC nodule needs further studies.

**Fig. 5:**
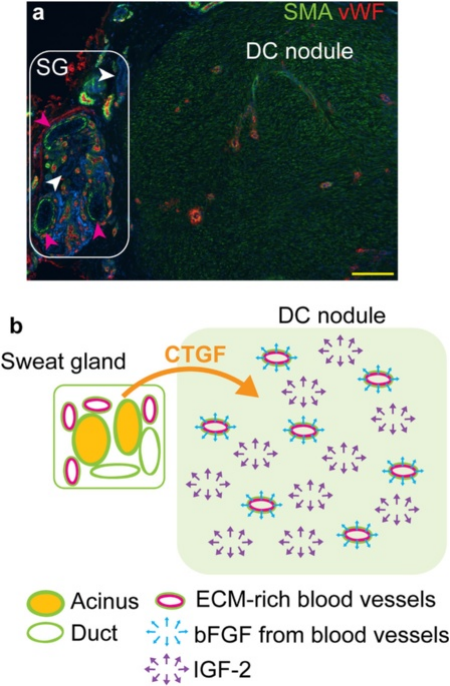
A schematic overview of growth factor signaling in DC. DC tissue was stained with antibodies recognizing SMA and vWF. Sweat glands (SG), which contain SMA-positive acini (a; *violet arrows*), SMA-negative ducts (a; *white arrows*) and SMA/vWF double-positive blood vessels are located adjacent to the SMA-rich DC nodule, which contains vWF-expressing blood vessels (**a**). Scale bar: 200 μm. bFGF synthetized by the endothelial cells and IGF-2 present throughout the DC tissue synergize in the ECM-rich myofibroblast layer of the blood vessels to form a supportive environment for sustained cell proliferation in the vascular and perivascular compartments. Sweat glands synthesize profibrotic CTGF, which, in combination with growth factors present in the DC tissue, facilitates the development of fibrosis (**b**). *bFGF* basic fibroblast growth factor, *CTGF* connective tissue growth factor, *DC* Dupuytren’s contracture, *EMC* extracellular matrix, *IGF-2* insulin-like growth factor 2, *SMA* smooth muscle actin, *vWF* von Willebrand’s factor

A few proproliferative growth factors have been implicated in the pathogenesis of DC. Blood vessels in DC nodules express high levels of basic fibroblast growth factor (bFGF) [10] and it has been proposed that persistent TGF-β signaling in conjunction with increased bFGF levels might be responsible for the increased proliferation and fibrosis taking place in DC [30]. IGF-2 has been shown to regulate cellular contractility and proliferation in DC [12] and recently it was suggested that CTGF, a TGF-β target gene and a potent activator of the AKT signaling pathway is implicated in the pathogenesis of fibrotic diseases including DC [11, 21]. CTGF is secreted by cultivated endothelial cells [31] and therefore we assumed that CD105/CD90-positive activated endothelium might synthesize this growth factor. Surprisingly, no CTGF signal was detected in the endothelial cells, however, the acinar cells of DC-associated sweat glands were positive for this molecule, which suggest that sweat glands might have an hitherto unrecognized role in the development of DC lesions by providing proproliferative profibrotic CTGF signaling to the DC tissue (Fig. 5a, b). Furthermore, the inhibition of CTGF might be a promising novel therapeutic approach in the DC treatment since corresponding inhibitors are available and are currently undergoing clinical trials as treatments for hematopoietic malignancies [21].

The composition of the ECM plays a critical role in the regulation of cell fate and regenerative potential [32]. We found that laminin subunits α4, α5, β1, β2, and γ1, which correspond to laminins 411/421 and 511/521, were enriched in the DC tissue. These laminins are normal components of the endothelial basement membrane [22] and as expected we found that the high laminin content of the DC nodule is caused by the increased vascularization.

## Conclusions

The somatic stem cells are often confined to stem cell niches - areas with specific microenvironment, which maintains their unique properties. It can be envisaged that the presence of supportive ECM molecules in conjunction with the presence of oxygen supply and nutrients create a favorable microenvironment in the vicinity of small blood vessels, which sustains the cell renewal in the DC nodule. Notably, laminins 511 and 521 have been shown to support the undifferentiated state of mouse and human embryonic stem cells (hESC) and keratinocytes [15, 16]. Furthermore, the cooperative action of IGF-2 and bFGF plays a crucial role in the establishment of the regulative niche for hESC [33], which sustains the pluripotent properties of hESC. This suggests that the combined action of bFGF and IGF-2 in the context of high laminin 511/521 content in the surrounding ECM might provide a supportive environment for the persistence of cells with enhanced proliferative potential also in the DC nodules.

## Additional files

Additional file 1: Table S1. Listing of the used antibodies and their dilutions; legends for Figures S1-S3. Additional file 2: Figure S1. Characterization of normal palmar fascia (NPF) and Dupuytren’s contracture (DC) samples in respect of endothelial, myofibroblast and proliferation markers. Additional file 3: Figure S2. Laminins 411/421 and 511/521 are highly expressed in the Dupuytren’s contracture (DC) tissue. Additional file 4: Figure S3. Expression of laminin subunits and fibronectin in the Dupuytren’s contracture (DC) and normal palmar fascia (NPF) tissue.

## Abbreviations

AKT: Ak mouse strain thymoma-associated
bFGF: basic fibroblast growth factor
cDNA: complementary DNA
CoIV: collagen IV
CTGF: connective tissue growth factor
DC: Dupuytren’s contracture
ECM: extracellular matrix
FITC: fluorescein isothiocyanate
FN: fibronectin
hESC: human embryonic stem cells
IGF-2: insulin-like growth factor 2
K15: cytokeratin 15
MAPK: mitogen-activated protein kinase
NPF: normal palmar fascia
pAKT: phosphorylated AKT
PBS: phosphate-buffered saline
qPCR: quantitative polymerase chain reaction
SMA: smooth muscle actin
TGF-β: transforming growth factor β
vWF: von Willebrand’s factor

## References

[1] Rayan GM (2007). Dupuytren disease: Anatomy, pathology, presentation, and treatment. J Bone Joint Surg Am.

[2] Dibenedetti DB, Nguyen D, Zografos L, Ziemiecki R, Zhou X (2011). Prevalence, incidence, and treatments of Dupuytren’s disease in the United States: results from a population-based study. Hand (N Y).

[3] Rehman S, Goodacre R, Day PJ, Bayat A, Westerhoff HV. Dupuytren’s: a systems biology disease. Arthritis Res Ther. 2011;13:238. doi:10.1186/ar3438, ar3438. 10.1186/ar3438 PMC3308066 21943049

[4] Shih B, Bayat A (2010). Scientific understanding and clinical management of Dupuytren disease. Nat Rev Rheumatol.

[5] Shaw Jr RB, Chong AK, Zhang A, Hentz VR, Chang J. Dupuytren’s disease: history, diagnosis, and treatment. Plast Reconstr Surg. 2007;120:44e–54e. doi:10.1097/01.prs.0000278455.63546.03. 10.1097/01.prs.0000278455.63546.03 17700106

[6] Shih B, Wijeratne D, Armstrong DJ, Lindau T, Day P, Bayat A (2009). Identification of biomarkers in Dupuytren’s disease by comparative analysis of fibroblasts versus tissue biopsies in disease-specific phenotypes. J Hand Surg Am.

[7] Berndt A, Kosmehl H, Katenkamp D, Tauchmann V (1994). Appearance of the myofibroblastic phenotype in Dupuytren’s disease is associated with a fibronectin, laminin, collagen type IV and tenascin extracellular matrix. Pathobiology.

[8] Kraljevic Pavelic S, Sedic M, Hock K, Vucinic S, Jurisic D, Gehrig P (2009). An integrated proteomics approach for studying the molecular pathogenesis of Dupuytren’s disease. J Pathol.

[9] Krause C, Kloen P, Ten Dijke P (2011). Elevated transforming growth factor beta and mitogen-activated protein kinase pathways mediate fibrotic traits of Dupuytren’s disease fibroblasts. Fibrogenesis Tissue Repair.

[10] Gonzalez AM, Buscaglia M, Fox R, Isacchi A, Sarmientos P, Farris J (1992). Basic fibroblast growth factor in Dupuytren’s contracture. Am J Pathol.

[11] Satish L, Gallo PH, Baratz ME, Johnson S, Kathju S (2011). Reversal of TGF-beta1 stimulation of alpha-smooth muscle actin and extracellular matrix components by cyclic AMP in Dupuytren’s-derived fibroblasts. BMC Musculoskelet Disord.

[12] Raykha C, Crawford J, Gan BS, Fu P, Bach LA, O'Gorman DB (1832). IGF-II and IGFBP-6 regulate cellular contractility and proliferation in Dupuytren’s disease. Biochim Biophys Acta.

[13] Durbeej M (2010). Laminins. Cell Tissue Res.

[14] Domogatskaya A, Rodin S, Boutaud A, Tryggvason K (2008). Laminin-511 but not −332, −111, or −411 enables mouse embryonic stem cell self-renewal in vitro. Stem Cells.

[15] Rodin S, Domogatskaya A, Strom S, Hansson EM, Chien KR, Inzunza J (2010). Long-term self-renewal of human pluripotent stem cells on human recombinant laminin-511. Nat Biotechnol.

[16] Li A, Pouliot N, Redvers R, Kaur P (2004). Extensive tissue-regenerative capacity of neonatal human keratinocyte stem cells and their progeny. J Clin Invest.

[17] Kosmehl H, Berndt A, Katenkamp D, Mandel U, Bohle R, Gabler U, et al. Differential expression of fibronectin splice variants, oncofetal glycosylated fibronectin and laminin isoforms in nodular palmar fibromatosis. Pathol Res Pract. 1995;191:1105–13. doi:10.1016/S0344-0338(11)80655-2. 10.1016/S0344-0338(11)80655-2 8822112

[18] Spitzer M, Wildenhain J, Rappsilber J, Tyers M. BoxPlotR: a web tool for generation of box plots. http://boxplot.tyerslab.com. 10.1038/nmeth.2811 PMC3930876 24481215

[19] Nassiri F, Cusimano MD, Scheithauer BW, Rotondo F, Fazio A, Yousef GM (2011). Endoglin (CD105): a review of its role in angiogenesis and tumor diagnosis, progression and therapy. Anticancer Res.

[20] Schubert K, Polte T, Bonisch U, Schader S, Holtappels R, Hildebrandt G (2011). Thy-1 (CD90) regulates the extravasation of leukocytes during inflammation. Eur J Immunol.

[21] Lu H, Kojima K, Battula VL, Korchin B, Shi Y, Chen Y (2014). Targeting connective tissue growth factor (CTGF) in acute lymphoblastic leukemia preclinical models: anti-CTGF monoclonal antibody attenuates leukemia growth. Ann Hematol.

[22] Yousif LF, Di Russo J, Sorokin L (2013). Laminin isoforms in endothelial and perivascular basement membranes. Cell Adh Migr.

[23] Howard JC, Varallo VM, Ross DC, Faber KJ, Roth JH, Seney S (2004). Wound healing-associated proteins Hsp47 and fibronectin are elevated in Dupuytren’s contracture. J Surg Res.

[24] Calabrese C, Poppleton H, Kocak M, Hogg TL, Fuller C, Hamner B (2007). A perivascular niche for brain tumor stem cells. Cancer Cell.

[25] Paul G, Ozen I, Christophersen NS, Reinbothe T, Bengzon J, Visse E (2012). The adult human brain harbors multipotent perivascular mesenchymal stem cells. PLoS One.

[26] Wandel E, Saalbach A, Sittig D, Gebhardt C, Aust G (2012). Thy-1 (CD90) is an interacting partner for CD97 on activated endothelial cells. J Immunol.

[27] Burrows FJ, Derbyshire EJ, Tazzari PL, Amlot P, Gazdar AF, King SW (1995). Up-regulation of endoglin on vascular endothelial cells in human solid tumors: implications for diagnosis and therapy. Clin Cancer Res.

[28] Marioni G, D'Alessandro E, Giacomelli L, Staffieri A (2010). CD105 is a marker of tumour vasculature and a potential target for the treatment of head and neck squamous cell carcinoma. J Oral Pathol Med.

[29] Wikstrom P, Lissbrant IF, Stattin P, Egevad L, Bergh A (2002). Endoglin (CD105) is expressed on immature blood vessels and is a marker for survival in prostate cancer. Prostate.

[30] Baird KS, Crossan JF, Ralston SH (1993). Abnormal growth factor and cytokine expression in Dupuytren’s contracture. J Clin Pathol.

[31] Bradham DM, Igarashi A, Potter RL, Grotendorst GR (1991). Connective tissue growth factor: a cysteine-rich mitogen secreted by human vascular endothelial cells is related to the SRC-induced immediate early gene product CEF-10. J Cell Biol.

[32] Watt FM, Huck WT (2013). Role of the extracellular matrix in regulating stem cell fate. Nat Rev Mol Cell Biol.

[33] Bendall SC, Stewart MH, Menendez P, George D, Vijayaragavan K, Werbowetski-Ogilvie T (2007). IGF and FGF cooperatively establish the regulatory stem cell niche of pluripotent human cells in vitro. Nature.

