# Supplementary material for: Laminin-rich blood vessels display activated growth factor signaling and act as the proliferation centers in Dupuytren’s contracture
Source: Arthritis Res Ther. 2015 May 28;17(1):144. doi: 10.1186/s13075-015-0661-y (PMC4475288; doi:10.1186/s13075-015-0661-y)
Supplement: Additional file 1: Table S1. — Listing of the used antibodies and their dilutions; legends for Figures S1-S3. [file 13075_2015_661_MOESM1_ESM.pdf]

Supplementary information.

Laminin-rich blood vessels display activated growth factor signaling and act as the proliferation centers in Dupuytren's contracture.

Authors

Janeli Viil[1], Katre Maasalu[2,3], Liis Tamming[1], Kadi Lõhmussaar[1], Mikk Tooming[1], Sulev Ingerpuu[1], Aare Märtson[2,3] and Viljar Jaks[1*]

Affiliations

[1]Institute of Molecular and Cell Biology, University of Tartu, Tartu, Estonia.

[2]Department of Traumatology and Orthopedics, University of Tartu, Tartu, Estonia.

[3]Clinic of Traumatology and Orthopedics, Tartu University Hospital, Tartu, Estonia.

*Corresponding author:

Viljar Jaks, PhD;

Institute of Molecular and Cell Biology, University of Tartu

Riia 23, 51010

Tartu, Estonia

Tel: +372 7374 069

e-mail: viljar.jaks@ut.ee

Supplementary figure legends

Fig. S1. Characterization of normal palmar fascia (NPF) and Dupuytren's contracture (DC) samples in respect of endothelial, myofibroblast and proliferation markers. The NPF and DC samples were stained with antibodies recognizing CD73, CD90, CD105, von Willebrand Factor (vWF), smooth muscle actin (SMA) and proliferation marker Ki67 as indicated on the panels. No Ki67-positive proliferating cells were found in NPF samples (a). Note that the nonspecific background signal in green channel is deliberately amplified to underline the absence of specific signal for Ki67. SMA and vWF label small blood vessels in DC tissue (b). NPF samples contain only rarely blood vessels (c) while in DC samples numerous SMA-labeled blood vessels can be found (d). Note that the myofibroblast layer surrounding blood vessels was thicker in DC samples than in NPF samples (c, d). CD105/CD90 double-positive cells localize in blood vessels in the DC tissue (e); CD105 is expressed at the apical compartment of the vWF-positive endothelial cells (f); rare vWF-positive blood vessels found in the NPF samples were devoid of CD105 expression (g). Note that the nonspecific background signal in green channel is deliberately amplified to underline the absence of specific signal for CD105. SMA-expressing myofibroblasts encompass the CD90-positive endothelial cells in DC (h) and only rare CD90-expressing cells could be found in the NPF tissue (i). No CD105/CD73 double positive cells could be identified in DC (j). Scale bars: a, b, d - 200 µm; c, e, g, j - 50 µm; f, h, i - 20 µm.

Fig. S2. Laminins 411/421 and 511/521 are highly expressed in the DC tissue. DC and NPF samples were stained with antibodies recognizing laminin subunits α4, α5, β1, β2 and γ1 as indicated on the panels. All the tested laminin subunits were expressed in the DC samples (a-e). None of these laminin subunits was detected in the NPF samples (f-j). Scale bars: 200 µm.

Fig. S3. Expression of laminin subunits and fibronectin in the DC and NPF tissue. DC and NPF samples were stained with antibodies recognizing laminin subunits α2, α3, α5, β3, γ2, collagen IV (CoIV) and fibronectin (FN) as indicated on the panels. The expression of laminin subunits α2, α3, β3, and γ2 was not detected in the DC (a-d) and NPF (e-h) samples. The expression of laminin subunits α4, α5, β1, β2 and γ1 was overlapping with collagen IV expression showing the presence of functional basement membrane (i-m). FN deposition was substantially increased in the ECM of the DC tissue (o) when compared to the NPF (n). A considerable amount of FN was localized in the laminin α5-labeled perivascular compartment in the DC tissue (p). DAPI was used to counterstain nuclei. Scale bars: 200 µm.

Supplementary Table 1.

List of primary antibodies.

Antibody	Host, clone/cat no	Dilution/ final concentration	Source, reference
Laminin alpha 2	Mouse, (B-4), sc-55605	1:50	SCBT*
Laminin alpha 3	Rabbit, (H187), sc-20143	1:50	SCBT*
Laminin alpha 4	Mouse, clone 3H2	0,2 mg/ml	(Wondimu et al. 2004)
Laminin alpha 5	Mouse, clone 4B5	0,1 mg/ml	(Wondimu et al. 2013)
Laminin beta 1	Mouse, clone DG10	0,02 mg/ml	(Nigatu et al. 2006)
Laminin beta 2	Mouse, (C4),sc-59980	1:100	SCBT*
Laminin beta 3	Rabbit, (H-300), sc-20775	1:50	SCBT*
Laminin gamma 1	Mouse, clone 2E8, MAB1920	1:30	Millipore
Laminin gamma 2	Rabbit, (H-183), sc-20776	1:100	SCBT*
Laminin gamma 3	Rabbit, SAB4500081	1:100	Sigma-Aldrich
CD105-FITC	Mouse, (MEM-229), ab53318	1:100	Abcam
Collagen IV α2	Rabbit, C12196	1:200	AssaybioTech
Cytokeratin 15	Mouse, LHK15, ab80522	1:50	Abcam
FGF-2	Mouse, (6), sc-136255	1:100	SCBT*
Fibronectin	Rabbit, ab2413	1:200	Abcam
IGF-II	Goat, AF-292-NA	0,165 mg/ml	R&D Systems
Phospho-Akt (Thr308)	Rabbit, (C31E5E), #2965	1:800	Cell Signaling Technology
CTGF	Goat, (L-20), sc-14939	1:50	SCBT*
Ki67	Rabbit, NCL-Ki67p	1:500	Leica
Ki67	Rabbit, clone ID EPR3611, 2642-1	1:500	Epitomics
Smooth Muscle Actin	Mouse, clone αsm-1, NCL-SMA	1:50	Leica

Smooth Muscle Actin	Rabbit, clone EPR5368, 5264-1	1:2000	Epitomics
von Willebrand Factor	Rabbit, A0082	1:2000	Dako
Desmin	Mouse, clone D33, M0760	1:50	Dako
CD90 (Thy-1)	Mouse, clone F15-42-1, CBL415	1:50	Millipore
CD73	Mouse, clone AA60-E3-3, MABD122	1:50	Millipore
Mouse IgG	sc-2025	1:200	SCBT*

* SCBT = Santa Cruz Biotechnology

Supplementary references

Nigatu A, Sime W, Gorfu G, Geberhiwot T, Anduren I, Ingerpuu S, Doi M, Tryggvason K, Hjemdahl P, Patarroyo M (2006) Megakaryocytic cells synthesize and platelets secrete alpha5-laminins, and the endothelial laminin isoform laminin 10 (alpha5beta1gamma1) strongly promotes adhesion but not activation of platelets. Thromb Haemost 95:85-93

Wondimu Z, Geberhiwot T, Ingerpuu S, Juronen E, Xie X, Lindbom L, Doi M, Kortesmaa J, Thyboll J, Tryggvason K, Fadeel B, Patarroyo M (2004) An endothelial laminin isoform, laminin 8 (alpha4beta1gamma1), is secreted by blood neutrophils, promotes neutrophil migration and extravasation, and protects neutrophils from apoptosis. Blood 104:1859-1866

Wondimu Z, Omrani S, Ishikawa T, Javed F, Oikawa Y, Virtanen I, Juronen E, Ingerpuu S, Patarroyo M (2013) A novel monoclonal antibody to human laminin alpha5 chain strongly inhibits integrin-mediated cell adhesion and migration on laminins 511 and 521. PLoS One 8:e53648
