# Supplementary figures and images for: Laminin-rich blood vessels display activated growth factor signaling and act as the proliferation centers in Dupuytren’s contracture
Source: Arthritis Res Ther. 2015 May 28;17(1):144. doi: 10.1186/s13075-015-0661-y (PMC4475288; doi:10.1186/s13075-015-0661-y)

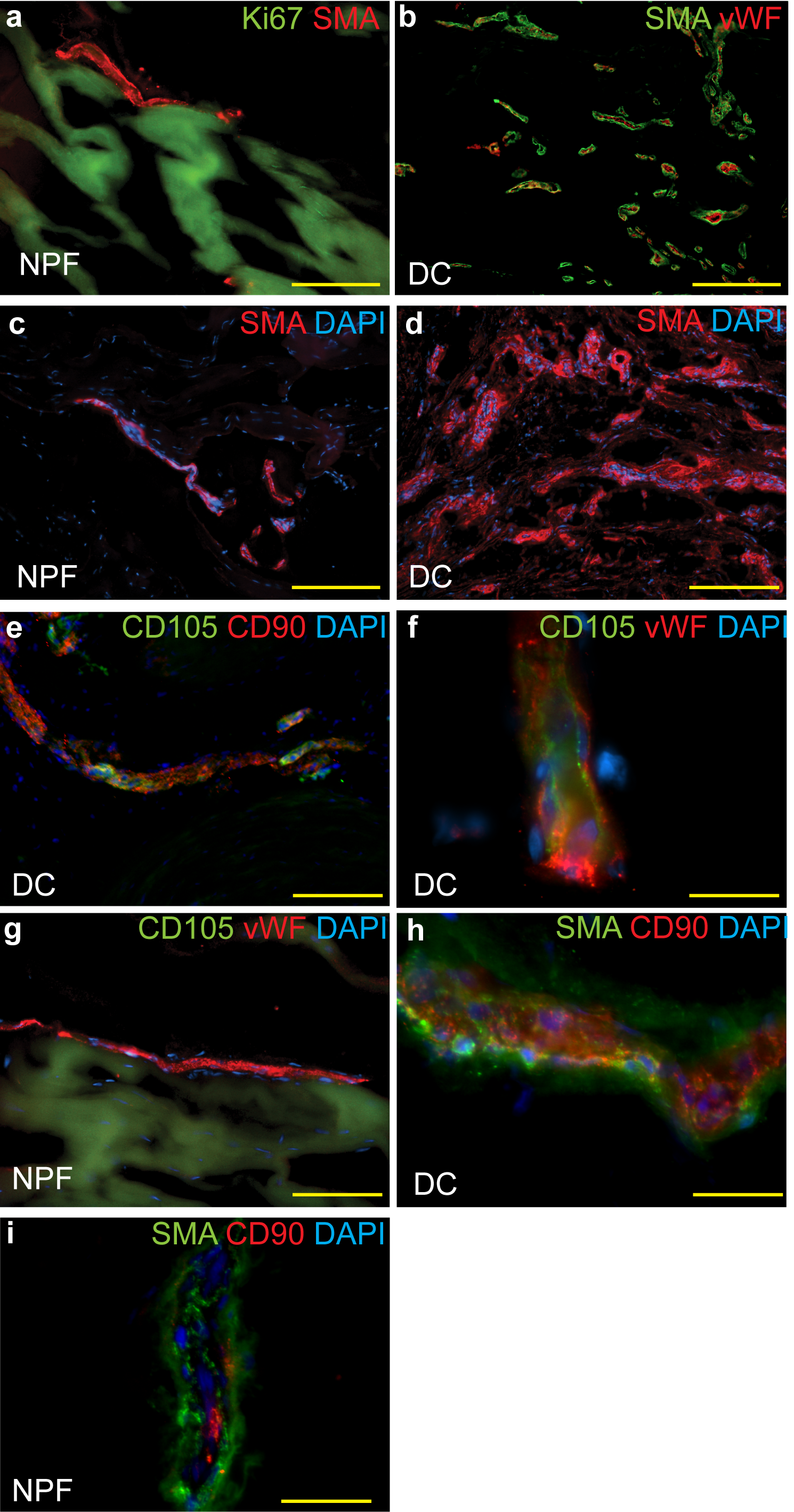

Supplement: Additional file 2: Figure S1. — Characterization of normal palmar fascia (NPF) and Dupuytren’s contracture (DC) samples in respect of endothelial, myofibroblast and proliferation markers. [file 13075_2015_661_MOESM2_ESM.tiff]

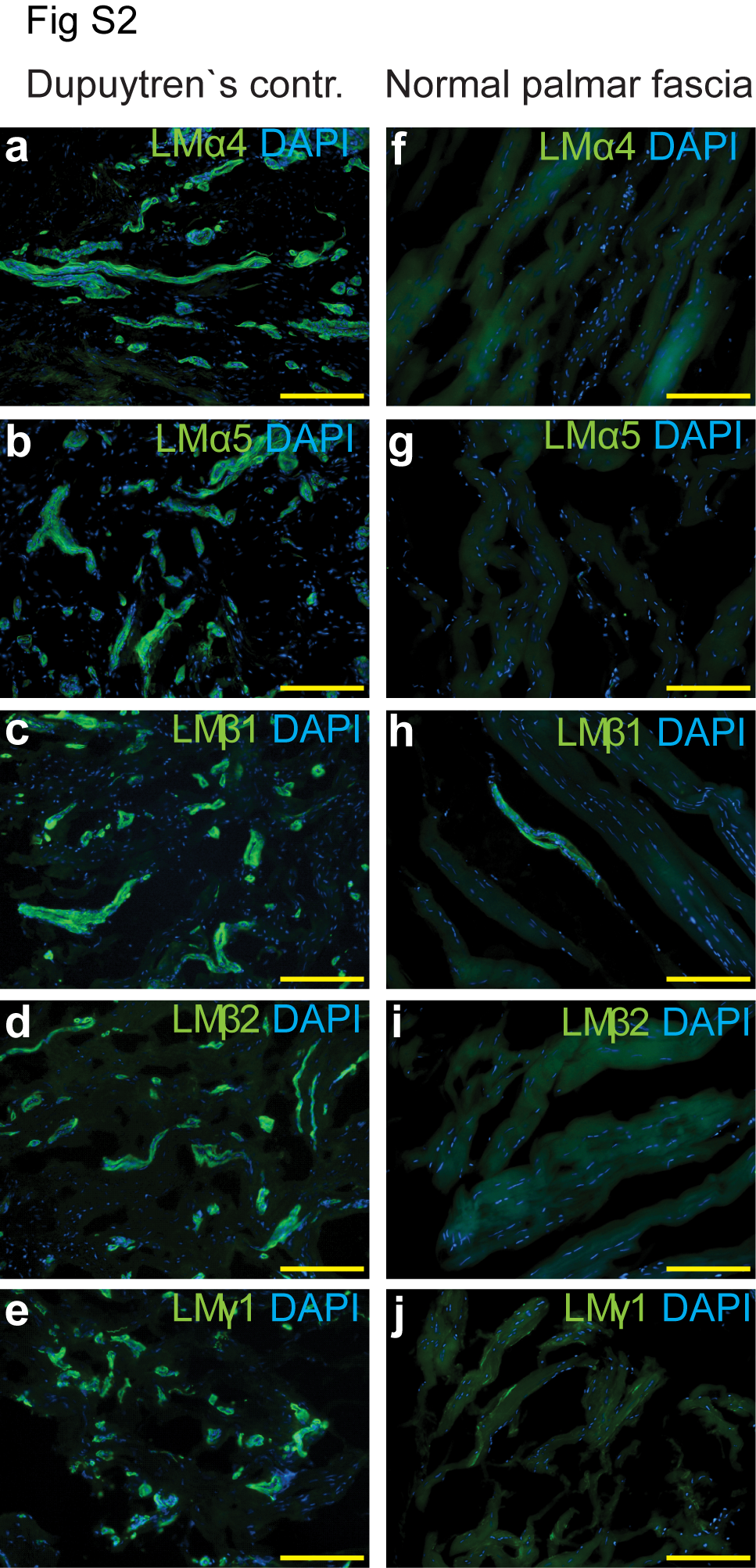

Supplement: Additional file 3: Figure S2. — Laminins 411/421 and 511/521 are highly expressed in the Dupuytren’s contracture (DC) tissue. [file 13075_2015_661_MOESM3_ESM.tiff]

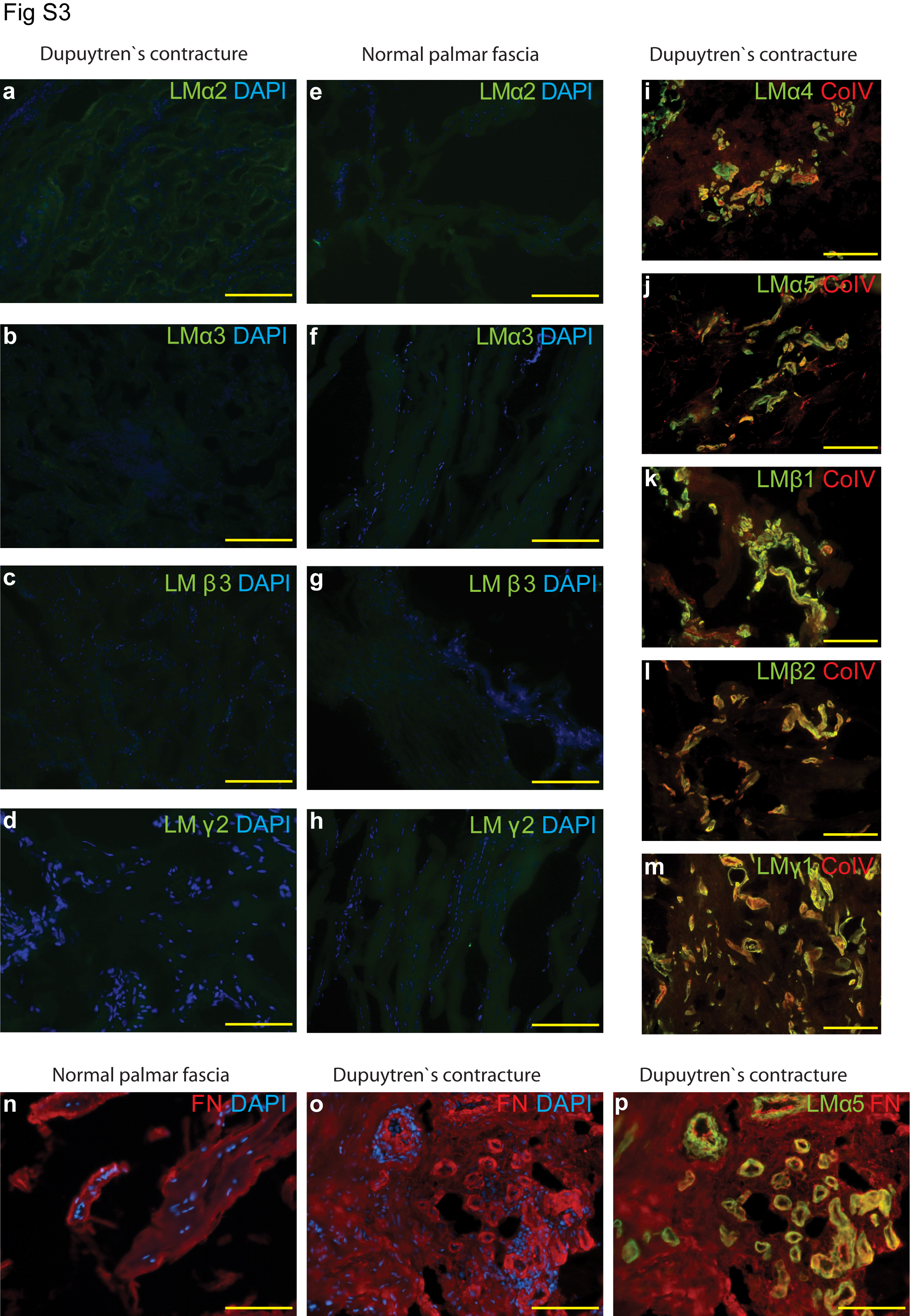

Supplement: Additional file 4: Figure S3. — Expression of laminin subunits and fibronectin in the Dupuytren’s contracture (DC) and normal palmar fascia (NPF) tissue. [file 13075_2015_661_MOESM4_ESM.tiff]
